# Digital Participatory Surveillance and the Zika Crisis: Opportunities and Caveats

**DOI:** 10.1371/journal.pntd.0004795

**Published:** 2016-06-13

**Authors:** Claudia Pagliari, Santosh Vijaykumar

**Affiliations:** 1 Edinburgh Global Health Academy, Usher Institute of Population Health Sciences and Informatics, University of Edinburgh Medical School, Edinburgh, United Kingdom; 2 Emerging Technology Lab, School of Computer Science and Engineering, Nanyang Technological University, Singapore; Imperial College London, UNITED KINGDOM

## Introduction

Managing the global threat of Zika requires innovative solutions. This article examines the potential of Digital Participatory Surveillance to support the management of global disease outbreaks by enabling citizens to report signs of infection. We discuss the status of the current evidence-base, contextual factors influencing user engagement and data quality, challenges for evaluation, and unique aspects of Zika with implications for design. We also suggest priorities for research, development and practice, to help translate the theoretical benefits of these methods into meaningful improvements in outbreak monitoring and public health.

## Zika and the Need for Novel Approaches

**Fig 1:**
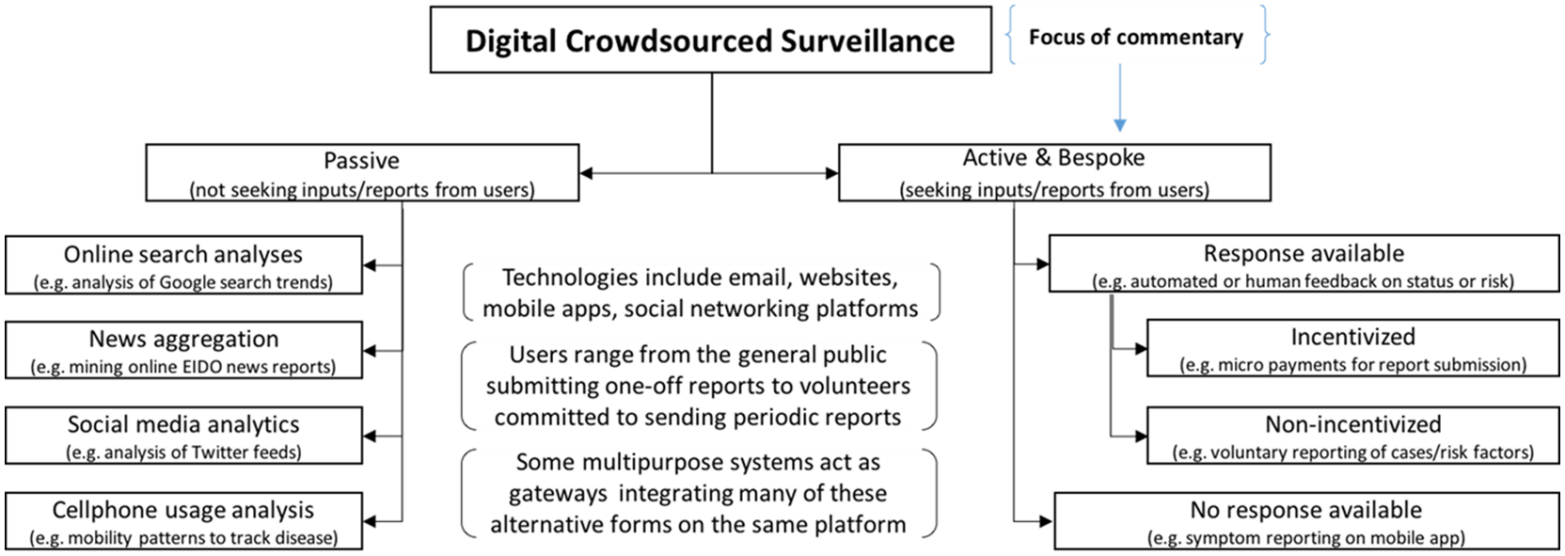
Types of digitally crowdsourced infectious disease surveillance.

As Zika turns from a ‘mild medical curiosity’ into a global public health crisis, finding new ways of predicting and monitoring its spread is becoming increasingly vital. The limitations of traditional infectious disease surveillance methods and increased use of the internet and mobile phones have inspired innovative approaches for crowdsourcing indicators from members of the public. Many of these rely on the secondary analysis of people’s Internet, social media or cell phone activity, sometimes called infoveillance [1]. Others involve the proactive reporting of symptoms, signs and risk factors by citizens and volunteers (see Fig 1). This commentary considers the latter category, which we refer to as Digital Participatory Surveillance (DPS). Concerted DPS efforts typically require participants to register and submit one-off or periodic reports using a structured form. These reports can be aggregated, analysed and summarised—often via dynamic geo-tagged disease maps—to notify public health agencies, practitioners and communities, enable risk prediction and inform preventive and responsive interventions. The potential of DPS to augment traditional outbreak monitoring has inspired much enthusiasm within the global health community, along with investments on the part of donors and governments. In contrast, the quality and effectiveness of these methods has received far less coverage. Given the immediate global health threat posed by Zika it is timely to consider the role and value of DPS in Emerging and Infectious Disease Outbreaks (EIDO), in order to inform policy, practice and research.

## Effectiveness of Digital Participatory Surveillance

Recent literature reviews [2] and examination of information available on DPS project websites reveal that the majority of existing DPS research concerns influenza in high income countries, although comparable endemic tropical diseases such as Dengue fever have also been studied [3]. Such research chiefly examines the magnitude and characteristics of public participation [4], disease incidence rates measured via DPS compared with traditional surveillance [5] and use of DPS data to identify predictors of illness or retrospectively measure prevalence [6]. This research has been important for understanding the potential reach, timeliness, accuracy and reliability of DPS as a complement to traditional surveillance efforts. However, evidence of substantive outcomes for public health remains sparse. Studies evaluating DPS projects in EIDO of international concern, such as Zika, are poorly represented in the academic literature, arguably reflecting the priority of response over planned research in such circumstances. Thus although there has been much activity; most recently surrounding Ebola; the impacts of DPS on stakeholders’ behaviour, institutional responses and population health in EIDO remain largely unknown.

## Challenges for Evaluation

One explanation for this lack of evidence is the difficulty involved in evaluating the effects of DPS initiatives. For one thing, their users tend to be geographically scattered, and may be anonymous, making it hard to judge the quality, timeliness or representativeness of the data being returned, particularly when the number of participants is very low relative to the size of the population or area. Undertaking evaluation in the high pressure context of EIDO can also be challenging, with new outbreaks, symptoms and environmental influences often arising in dynamic and uncontrollable ways. Deciding what to evaluate can present a dilemma for decision makers lacking guidance on prioritisation. Evaluation outcomes could range from the volume and content of information submitted, its correspondence with facility-based and infoveillance measures and its role in initiating or accelerating responses, to the influence of DPS on disease prevention, control or population health outcomes. As a result, many evaluation projects are retrospective and opportunities for rapidly translating research insights to action are lacking. Developing real-time evaluation metrics and tracking mechanisms is possible, given evolving ‘big data’ analytic techniques [7], but in the context of EIDO this may be easier said than done. Further work is also needed to understand how to measure the performance of DPS systems during EIDOs and define what constitutes success or failure.

## Factors Affecting Public Engagement

Poor adoption of DPS is a significant threat to their value as sentinel technologies, and raising citizens’ awareness of and engagement with such projects is vital, as is encouraging their persistence where regular reports are required. This engagement can be affected by a host of socio-contextual factors, such as users’ immediate priorities for care-seeking, cultural norms, stigma and superstition, technology preferences, media-inflated risk-perceptions, lack of institutional trust, or illiteracy [8–10]. In the case of Zika these issues are being compounded by a highly charged information climate, laden with public fear and multiple conspiracy theories [11]. Anecdotal accounts also suggest that in, some natural disasters, over-reporting of disease cases via DPS has been used as a strategy to leverage aid. Such influences can affect the representativeness, validity and usefulness of the data captured by DPS systems, as well as impeding their integration within coordinated regional information strategies for EIDO, but they remain poorly understood. Understanding the value of different DPS recruitment strategies is also essential; for example, in a study conducted in Europe and New Zealand, online approaches were less successful than traditional ones [12].

## Disease-Sensitive Design

The nature of the disease also has a bearing on what DPS approaches are likely to be most appropriate. In contrast to the horrific and deadly presentation of Ebola, symptoms of Zika in adults are usually mild and easily confused with other illnesses, while the critical health outcomes may not be seen until a child with microcephaly is born to an infected mother. This may be some months away, suggesting that different methods of recruitment, or perhaps peer-triage, may be required to obtain the most valid data [13]. The emphasis given to supporting expectant and would-be mothers in the context of Zika also contrasts with the prioritization of victim quarantine in Ebola, with different implications for compassionate or coercive public health and the types of DPS components that may be needed (e.g. tools for ‘reaching-out’ or ‘calling-out’). They also raise issues for human rights (e.g. Zika data may be used to pressure women into delaying pregnancy) and privacy (e.g. being identified from an Ebola posting could place a DPS participant at serious social or physical risk) [8], which call for reciprocal information and support, alongside robust security protocols. For example, the Mo-Buzz project; focused on Dengue fever; combined public reporting of suspected mosquito breeding sites with health communication and risk prediction [3]. The association of Zika with Guillain-Barré syndrome also calls for blended approaches that target men as well as women.

## The Need for Research and Innovation

The current Zika outbreak presents new opportunities for DPS to demonstrate its value in the context of EIDO in a variety of ways. These include informing rapid and targeted interventions, smarter health-aware transport management, anticipatory service planning, and minimising cross-border transmission. However, making best use of DPS for public health requires further understanding in a number of key areas. Importantly there is a need to refine our theories of how DPS will be used, and the pathways through which they will generate benefits, in order to enable more informed planning and interpretation of observed effects. Research is required to understand the usefulness of DPS under different circumstances and the effects of socio-contextual influences on citizen engagement and data quality. Understanding how different communities engage with bespoke DPS systems alongside their personal networking tools is also important; for example the use of WhatsApp in the prediction of Zika has been described [14]. We also need to investigate how DPS data can be deployed for tracking disease without compromising human rights, and how to most effectively combine citizen reporting with information, support and other forms of surveillance. Methodological innovation is necessary to enable active DPS initiatives to be evaluated using cumulative real-world evidence, recognizing individual, institutional and societal outcomes, while project sponsors and implementers should consider evaluation as a core requirement rather than an add-on. Technological innovation is needed to adapt survey-based DPS systems for real-time mobile reporting by citizens, to develop approaches for actively soliciting DPS data via users’ preferred social media, and to find the best ways of integrating DPS with other surveillance and dissemination tools to support the information needs of different stakeholders (e.g. via portals such as promedmail.org). The emerging science and practice of ‘social machines’ (e.g. sociam.org) also offers important opportunities to study the synergies between citizen sensors, digital networking and web analytics for making best use of collective intelligence in the context of EIDO. At the policy level, closer coupling of funding streams for digital innovation and health services research may also help to address the apparent mismatch between projects and evidence.

## Implications for Global Health

Other forms of digital disease surveillance have shown promise in improving the timeliness of outbreak discovery and public health communications [15]. The lack of comprehensive, interdisciplinary evidence on the use and benefits of DPS systems for EIDO in low- and middle-income countries is somewhat surprising, considering that this approach was first proposed around a decade ago. Nonetheless, studies from high-income countries provide grounds for cautious optimism. Given the urgency of the Zika crisis and other emerging threats, such as the Yellow Fever epidemic in Angola, we strongly encourage governments and donors to support these initiatives. The decision to proceed with the 2016 Olympic Games in Brazil provides a further impetus for mobilizing these innovative citizen-centred surveillance techniques as part of the regional and global public health response. Integrating research and evaluation into such deployments is essential for informing future policies and practices, and these current crises also represent opportunities to strengthen the evidence-base.

## Key Messages

- Zika is spreading unpredictably and involving citizens in digital disease surveillance may aid worldwide prevention and control efforts
- Designing effective Digital Participatory Surveillance approaches requires an appreciation of context, culture and disease characteristics, to optimise recruitment and data quality
- Most evidence of the benefits of DPS comes from studies of influenza tracking in high income countries. Far less is known about its role and effectiveness in managing global Emerging Infectious Disease Outbreaks
- Investing in DPS initiatives is likely to be worthwhile but integrating research and evaluation is vital for informing public health policies and programmes
- The forthcoming Brazil Olympics and the emerging Yellow Fever epidemic in Angola offer timely opportunities for DPS mobilization and research
